# Surveillance to achieve malaria elimination in eastern Myanmar: a 7-year observational study

**DOI:** 10.1186/s12936-022-04175-w

**Published:** 2022-06-07

**Authors:** Jade D. Rae, Suphak Nosten, Ladda Kajeechiwa, Jacher Wiladphaingern, Daniel M. Parker, Jordi Landier, Aung Myint Thu, Hsa Dah, Aye Be, Win Cho Cho, K.’Nyaw Paw, Eh Shee Paw, Paw Bway Shee, Christ Poe, Chit Nu, Baw Nyaw, Julie A. Simpson, Angela Devine, Richard J. Maude, Ku Ler Moo, Myo Chit Min, May Myo Thwin, Saw Win Tun, François H. Nosten

**Affiliations:** 1 Shoklo Malaria Research Unit (SMRU), Mahidol-Oxford Tropical Medicine Research Unit (MORU), Faculty of Tropical Medicine, Mahidol University, Mae Sot, Thailand; 2 Mahidol-Oxford Tropical Medicine Research Unit (MORU), Faculty of Tropical Medicine, Mahidol University, Bangkok, Thailand; 3 Centre for Tropical Medicine and Global Health, Nuffield Department of Medicine, University of Oxford, Oxford, UK; 4 Population Health and Disease Prevention, University of California-Irvine, Irvine, CA USA; 5 Epidemiology and Biostatistics, University of California-Irvine, Irvine, CA USA; 6 IRD (Institut de Recherche Pour Le Developpement), Aix Marseille Univ, INSERM, SESSTIM, Aix Marseille Institute of Public Health, ISSPAM, Marseille, France; 7 Centre for Epidemiology and Biostatistics, Melbourne School of Population and Global Health, The University of Melbourne, Melbourne, Australia; 8 Global and Tropical Health Division, Menzies School of Health Research, Charles Darwin University, Darwin, Australia; 9 Harvard TH Chan School of Public Health, Harvard University, Boston, USA; 10 The Open University, Milton Keynes, UK

## Abstract

**Background:**

The collection and utilization of surveillance data is essential in monitoring progress towards achieving malaria elimination, in the timely response to increases in malaria case numbers and in the assessment of programme functioning. This paper describes the surveillance activities used by the malaria elimination task force (METF) programme which operates in eastern Myanmar, and provides an analysis of data collected from weekly surveillance, case investigations, and monitoring and evaluation of programme performance.

**Methods:**

This retrospective analysis was conducted using data collected from a network of 1250 malaria posts operational between 2014 and 2021. To investigate changes in data completeness, malaria post performance, malaria case numbers, and the demographic details of malaria cases, summary statistics were used to compare data collected over space and time.

**Results:**

In the first 3 years of the METF programme, improvements in data transmission routes resulted in a 18.9% reduction in late reporting, allowing for near real-time analysis of data collected at the malaria posts. In 2020, travel restrictions were in place across Karen State in response to COVID-19, and from February 2021 the military coup in Myanmar resulted in widescale population displacement. However, over that period there has been no decline in malaria post attendance, and the majority of consultations continue to occur within 48 h of fever onset. Case investigations found that 43.8% of cases travelled away from their resident village in the 3 weeks prior to diagnosis and 36.3% reported never using a bed net whilst sleeping in their resident village, which increased to 72.2% when sleeping away from their resident village. Malaria post assessments performed in 82.3% of the METF malaria posts found malaria posts generally performed to a high standard.

**Conclusions:**

Surveillance data collected by the METF programme demonstrate that despite significant changes in the context in which the programme operates, malaria posts have remained accessible and continue to provide early diagnosis and treatment contributing to an 89.3% decrease in *Plasmodium falciparum* incidence between 2014 and 2021.

**Supplementary Information:**

The online version contains supplementary material available at 10.1186/s12936-022-04175-w.

## Background

Between 2012 and 2020, the number of *Plasmodium falciparum* cases in the Greater Mekong Sub-region (GMS) decreased by an estimated 95% [1]. However, vigilance must be maintained to achieve the goal of *P. falciparum* elimination by the year 2030 and reduce the risk of malaria resurgence in areas that have achieved elimination.

To achieve *P. falciparum* elimination, programmes rely in part on village-based malaria posts or community health workers, to provide access to free early diagnosis and treatment, thus reducing onward transmission [2, 3]. Data collected by malaria posts, including the number of positive cases and the number of consultations, are crucial in the planning and timely delivery of necessary targeted interventions in areas of high *P. falciparum* burden [4] or in response to increases in incidence in low transmission settings, in the monitoring of intervention impact, and in responding to decreases in malaria post service uptake. However, the utility of the data depends on how quickly it is collected, analysed, and responded to [5, 6].

The data collected by malaria posts and by the programme during routine activities are considered passive surveillance and is supplemented by (1) active surveillance which includes case investigations and (2) monitoring and evaluation assessments of malaria post performance. As case numbers decline case investigations become increasingly important in identifying patterns of risk including travel history prior to diagnosis [7], occupation [8, 9], and behaviours including the use of long-lasting insecticidal nets (LLINs) [10]. This information can then be used to guide interventions such as screening and treatment, or the delivery of awareness campaigns to improve the treatment seeking behaviour of high-risk populations. In Karen State, Myanmar, the METF programme has operated a network of 1,250 malaria posts over the period commencing in 2014 and ending in 2021. This programme has achieved significant reductions in *P. falciparum* incidence between 2014 and 2020 [11] owing to its ability to provide widescale access to malaria post services, near real-time data collection, and the prompt targeted delivery of mass drug administration in communities with high prevalence [4]. This paper describes the surveillance activities used by the METF programme to monitor case numbers and the quality of malaria post services, and assesses the completeness and timeliness of surveillance data collected, as well as the performance of the malaria posts and malaria post workers over space and time.

## Methods

### Study design and setting

This retrospective, observational study uses surveillance data collected from a cohort of 1250 malaria posts supported by the METF programme between 2014 to 2021 to evaluate the completeness and timeliness of the surveillance data collected, and to assess the ability of malaria posts and malaria post workers to provide early access to diagnosis and treatment.

The METF programme operates in four townships (the third-level administrative division in Myanmar) of Karen State: Hpapun, Hlaingbwe, Kawkareik and Myawaddy, covering approximately 18,002 km^2^ and serving an estimated rural population of 350,000. Karen State is a non-government-controlled area, not covered or accessible by the national health system due to village remoteness, and the ongoing conflicts in eastern Myanmar. This has resulted in the METF programme acting as the primary provider of malaria services in Karen State, which remains one of the Myanmar states with the highest malaria incidence [1]. Limited information on the geography and demography of Karen State was available at the start of the programme, so geographic reconnaissance and village surveys were undertaken at the beginning of the programme which are detailed elsewhere [3].

Village accessibility varies across Karen State based on geography and road networks. Hpapun in the north is mountainous, heavily forested, and has limited available road networks resulting in longer travel times between villages compared with Hlaingbwe and Kawkareik where most human settlements are in flatter terrain and road networks allow for easier movement between villages. Myawaddy in the south is mountainous but it is easier to navigate between villages than it is in Hpapun.

### Malaria posts

**Fig. 1:**
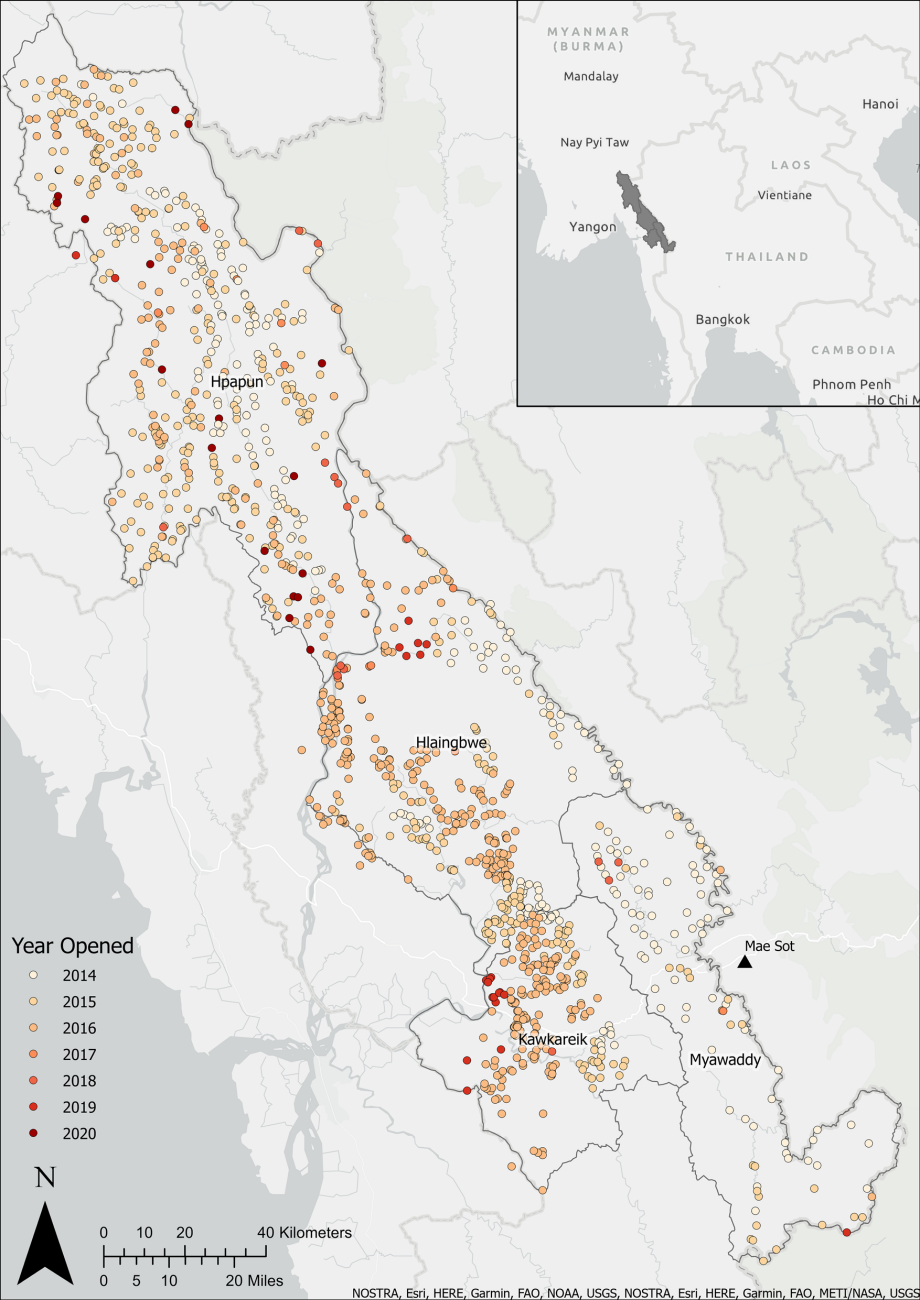
The complete malaria post network operated by the METF programme in four townships of Karen State, Myanmar. Each point represents a malaria post (coloured by year of opening), operated by a trained malaria post worker delivering free, uninterrupted access to diagnosis and treatment. Coordination of the programme and analysis of data is carried out in Mae Sot, Thailand (black triangle). Map generated using ArcGIS Pro version 2.5

In the first 3 years of the programme, the malaria post network expanded from 277 malaria posts in 2014 to 1,180 by the end of 2016. Malaria posts have been opened and closed based on village needs throughout the METF programme, with a total of 1250 malaria posts operating over the period commencing in 2014 and ending in 2021 (Fig. 1). Prior to the opening of each malaria post, meetings are held with villagers and village leaders to explain what malaria is, what services will be provided by the malaria post, and the importance of early diagnosis and treatment. At the village leader’s discretion an individual from the village is selected to perform the malaria post worker role, receiving 3 to 5 days of training on how to diagnose and treat cases of malaria, record information for each consultation, and maintain stocks of rapid diagnostic tests (RDTs) and medicines.

People with fever that present to a malaria post are tested using a *Plasmodium falciparum—Plasmodium vivax* rapid diagnostic test (RDT) (BiolineMalaria Ag P.f/P.v, Abbott Diagnostics Korea Inc). Cases of *P. falciparum* are treated with orally administered artemether–lumefantrine (artemether 5–24 mg/kg, lumefantrine 29–144 mg/kg) twice daily for three consecutive days, plus a single dose of primaquine (0.25 mg/kg) on the first day of treatment. Pregnant women diagnosed with *P. falciparum* in their first trimester are treated with orally administered Q7C7 (quinine 10 mg/kg and clindamycin 5 mg/kg) thrice daily for 7 days if it is their first diagnosis, or artemether–lumefantrine if they have already completed one round of Q7C7.

Cases of *P. vivax* are treated with chloroquine once daily for three consecutive days (10 mg/kg on days 1 and 2, 5 mg/kg on day 3). Treatment with an 8-aminoquinoline drug is necessary to eliminate the dormant liver stage (hypnozoites) of *P. vivax* parasites during infection, however due to the presence of glucose-6-phosphate-dehygrogenase (G6PD) deficiency in the Karen population, curative treatment of *P. vivax* is not administered due to the haemolytic effect of these drugs in G6PD deficient individuals [12, 13].

In the METF programme, Karen State is operationally organized into multiple administrative units, with Areas being the largest, Zones within the Areas (3 Areas divided into a total of 27 Zones), and with malaria posts organized by malaria post supervisor (roughly 9 malaria posts per supervisor, normally in a contiguous geographic area). Area coordinators oversee all activities conducted in their respective area, including the opening and closing of malaria posts, malaria post worker training, and targeted intervention delivery. Targeted interventions include mass drug administration (MDA) where everyone in the village receives *P. falciparum* treatment irrespective of diagnosis, described in detail elsewhere [4], and mass screen and treat (MSAT), where everyone in the village is tested and all positive individuals receive treatment. Planned activities are communicated to the Zone coordinators, who then organize meetings with villagers and village leaders to discuss the planned activities, and in the case of malaria post closure, present details on malaria post assessments and reason for closure.

Malaria post supervisors are responsible for ensuring adequate supplies of RDTs and medicine at their respective malaria posts, and for overseeing weekly reporting and the provision of malaria post worker salaries. Malaria post supervisors are selected by Zone coordinators and must have experience working in a health organization operating in Karen State. Malaria post supervisors receive the same training as the malaria post workers with additional training in the role of the supervisor. Monthly supervisor visits are performed to maintain regular contact between the malaria post worker and their supervisor and are conducted at the discretion of the malaria post supervisor.

All necessary malaria post supplies are paid for by the METF programme. The average monthly cost to operate a malaria post is 66.7 USD (excluding the cost of RDTs and anti-malarial medicines) which includes the monthly stipend of 41.8 USD for the malaria post worker (accounting for 62.7% of the total monthly costs), surveillance costs which include the collection and transport of data to the data entry sites (15.7 USD, 23.5%), and the structural maintenance of the malaria post (9.2 USD, 13.8%).

RDTs and anti-malarial medicines were procured by the United Nations Office for Project Services (UNOPS) through the pool procurement mechanism and the unit cost was not disclosed to the implementing partners. However, the market cost for RDTs is between 1 to 1.2 USD, and the artemisinin-based combination therapy used by the METF programme is between 1 to 1.5 USD per treatment.

The average cost per malaria post visit (during malaria post assessments, to deliver training or to conduct meetings with village leaders and villagers) is 67 USD which includes single or multiple modes of transport (i.e. boat, motorcycle, car, foot), accommodation in the village and a daily meal allowance.

### Data collection and management

#### Weekly surveillance data

Each week, malaria posts provide a summary of the number of consultations and the number of cases of *P. falciparum* and *P. vivax* diagnosed by age group and gender. For malaria posts in Hpapun, data are entered within 14 days from the end of the reporting week due to the absence of a reliable cell phone network. Hard copies of the data are transported to a local data entry site and are entered into an online data entry and management system (VooZaNoo). For malaria posts in Hlaingbwe, Kawkareik and Myawaddy, data are entered within 7 days from the end of the reporting week, where malaria posts rely on the cell phone network to transmit data via SMS to a cell phone at the local data entry site where data are extracted as an Excel file. The form used for weekly reporting is provided in Additional file 1.

Following data entry, weekly surveillance data are sent to the central METF office in Mae Sot, Thailand (Fig. 1) where data from the four townships are merged with data entered from previous weeks using Microsoft Access. Each week, the weekly data are cleaned and checked for inconsistencies between the number of tests, diagnoses and treatments delivered by gender and age, with data entry errors corrected following communication with the relevant data entry teams who review the raw data.

#### Consultation data

For each consultation at the malaria posts, patient information is collected including the date of diagnosis, gender, age, village of residence, pregnancy status for women, and the number of days since fever onset. Hard copies of the consultation data are sent to a local data entry site and are entered using Microsoft Access. Data are entered within 14 days from the end of the reporting week for malaria posts in Hpapun, where data are sent together with weekly surveillance data, and within 21 days from the end of the reporting week for malaria posts in Hlaingbwe, Kawkareik and Myawaddy, where hard copy data are transported less frequently.

#### Case investigation data

Case investigations are carried out in townships according to monthly case numbers. Case investigations began in 2017 with the investigation of *P. falciparum* cases diagnosed in Hlaingbwe, Kawkareik, and Myawaddy that could be contacted either directly or via the malaria post worker. With a decline in case numbers in 2019, case investigations were expanded to Hpapun.

During case investigations, information collected includes patient travel history (for travel out of the village in the 3 weeks prior to diagnosis), occupation, reason for delayed attendance to the malaria post (if more than 24 h after fever onset), and use of LLINs at home and during travel. The form used to collect information during case investigations is provided in Additional file 2.

#### Monitoring and evaluation data

From 2016, a subset of malaria posts across the four townships have been visited to assess their performance based on a set of pre-specified performance indicators including malaria post closure for more than 24 h, RDT and medicine stocks at the time of visit and in the previous month, and the number of supervisor visits. These indicators are classified as having either a minor or major impact on the ability of the malaria post to provide early access to diagnosis and treatment. The form used in the assessment of malaria post functioning is provided in Additional file 3.

The majority of monitoring and evaluation visits are conducted at malaria posts within travelling distance of malaria posts undergoing other surveillance activities, or at malaria posts that have not been visited recently. Targeted visits are also performed in response to possible issues in malaria post performance identified in surveillance data including low consultation rates, late reporting, or missing reports. Details on targeted monitoring and evaluation visits are provided elsewhere [11].

**Fig. 2:**
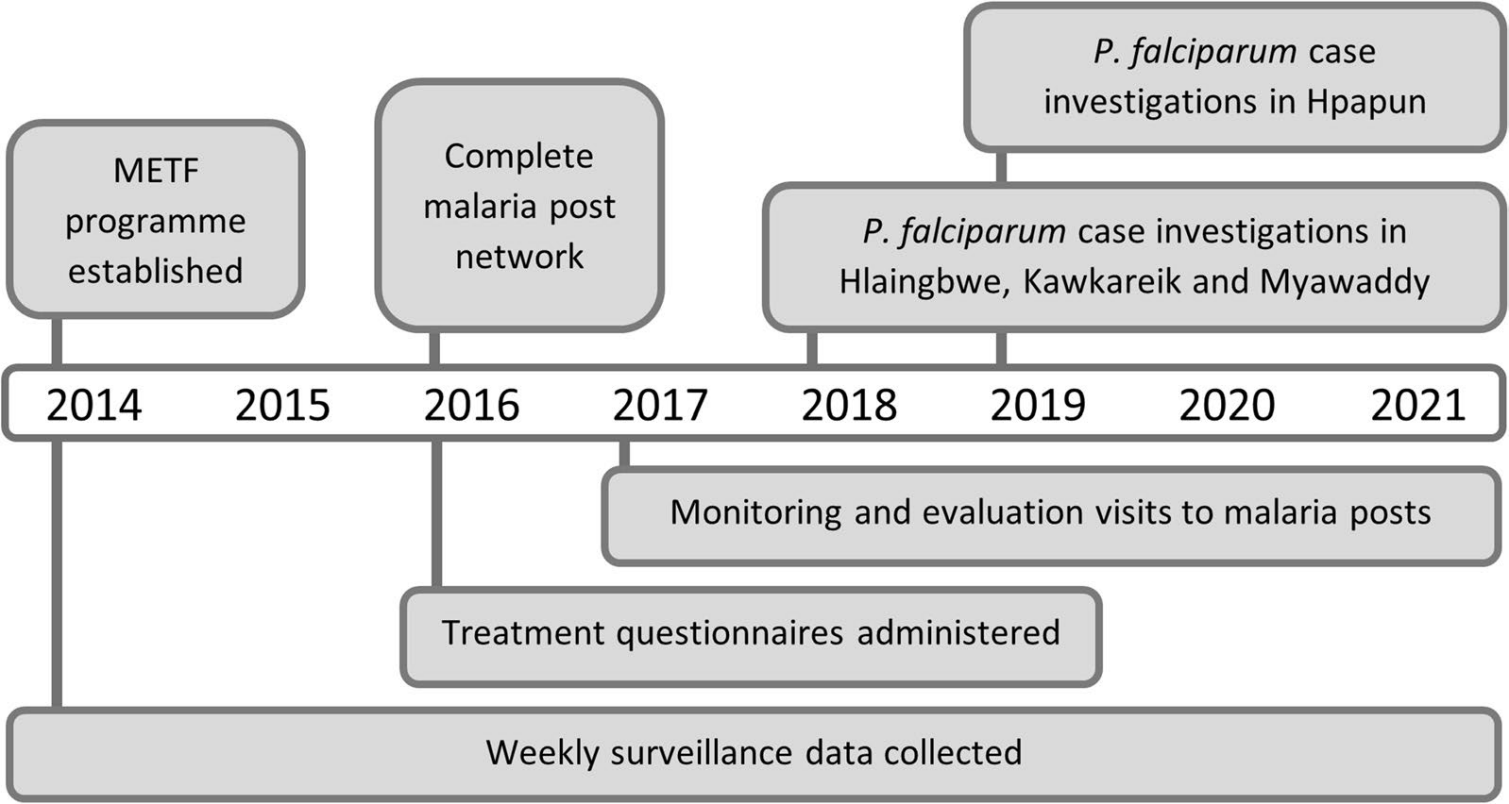
Timeline of the METF programme and surveillance activities. After the commencement of the METF programme in 2014 various targeted surveillance and performance assessments were conducted to maintain and assess the malaria post network performance

From 2016 to 2019, a questionnaire was used during monitoring and evaluation visits to assess malaria post worker treatment knowledge (Fig. 2). This questionnaire was also used opportunistically at training sessions and meetings where the knowledge of malaria post supervisors, and Area and Zone coordinators was also assessed. The questionnaire used to assess treatment knowledge is provided in Additional file 4.

In 2020 and 2021, as a result of COVID-19 travel restrictions and the military coup in Myanmar, treatment questionnaires could not be conducted, and the majority of case investigations and monitoring and evaluation visits were conducted remotely through cell phone contact with malaria post workers and malaria post supervisors.

### Statistical analysis

Data were summarized using the median, mean, proportion and percentage, to compare the functioning of the malaria posts, the quality of surveillance data collected, and case information between townships over time. Statistical analyses were performed using R (version 3.6) and mapping was performed in ArcGIS Pro (version 2.5).

## Results

### Weekly surveillance data

From May 2014 to November 2021, 371,564 weekly reports were received from 1250 malaria posts in the METF network. The date of data entry was recorded by the data entry team for 363,971 (97.9%) reports, of which 82.9% (301,741/363,971) were entered on time, within the 7-day (Hlaingbwe, Kawkareik or Myawaddy) or 14-day (Hpapun) time window.

In the first 3 years of the programme, the percentage of weekly reports received late reduced from 30.2% (1614/5351) in 2014 to 12.5% (6274/50151) in 2016. As a result of the military coup in Myanmar, which disrupted the usual data transmission routes, there was a 50.5% increase in late reporting in 2021 compared to 2020. Reports from Hpapun were the most impacted by these disruptions with 43.3% (8991/20766) of reports received late. Corresponding figures were 19.3% for Hlaingbwe, 15.1% for Myawaddy and 8.0% for Kawkareik. Additional file 5: Table S1 provides more details on weekly reports received late by year and township.

A total of 632 weekly reports were not received from the malaria posts between May 2014 and November 2021 inclusively, accounting for 0.2% of the total expected weekly reports. The majority of missing reports were from 2021 (92.6%, 585/632).

### Malaria post service uptake

Between May 2014 and November 2021, the METF malaria posts tested 678,717 cases of fever, of which 2.6% (17,744) tested positive for *P. falciparum*, 9.2% (62,538) tested positive for *P. vivax*, and 88.2% (598,435) tested negative for both. Additional file 5: Fig. S1 provides details on the gender and Additional file 5: Fig. S2 provides details on the age of *P. falciparum* and *P. vivax* cases over time.

**Fig. 3:**
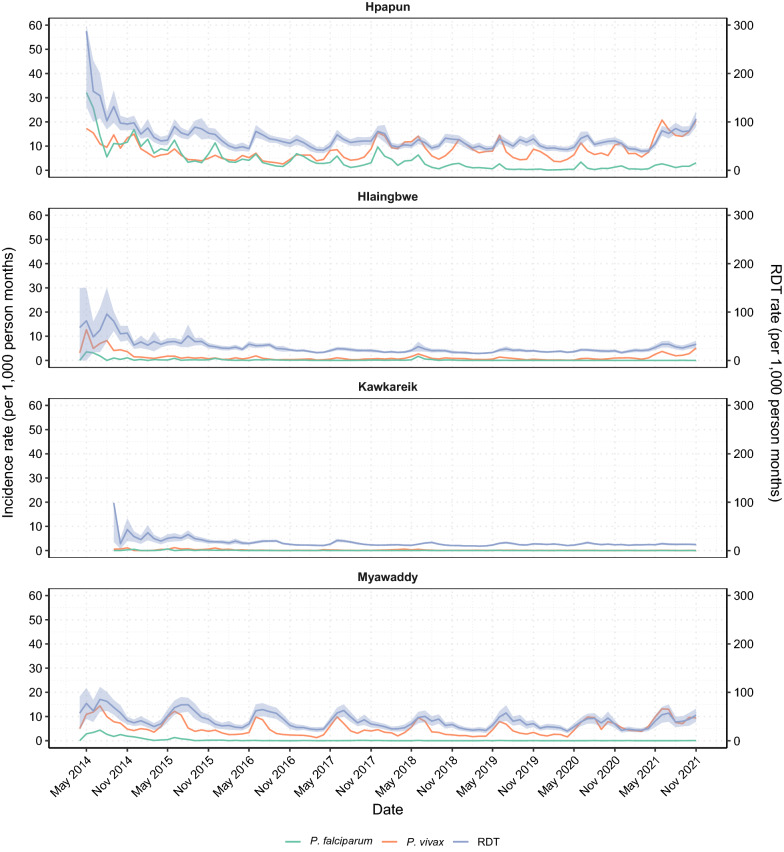
Average monthly rate of RDTs and malaria incidence by date and township. Average monthly rapid diagnostic testing rate (RDT—purple line) with 95% confidence intervals (purple area), and average monthly *P. falciparum* (green line) and *P. vivax* (orange line) incidence rates at the METF malaria posts

In 2021, the military coup in Myanmar resulted in the widescale displacement of people across Karen State in response to military attacks [15, 16], and the average monthly testing rate increased by 22.3% in Hpapun, 38.4% in Hlaingbwe and 13.7% in Myawaddy compared to 2020 (Fig. 3).

The majority of individuals were tested for malaria within 48 h of fever onset (53.2% within 24 h, and 28.2% between 24 and 48 h), 12.2% (78,729/640,961) were tested between 2 and 3 days, and 6.4% (40,978/640,961) were tested after more than 3 days of fever. Despite the COVID-19 pandemic, which resulted in village lockdowns and restricted travel (14), the average monthly testing rates remained stable from 2019 to 2020 across the four townships.

Information on village of residence was recorded for 98.0% (664,894) of fever cases tested, of which 90.9% of individuals were a resident of the village in which they were tested. By programme year this percentage increased from 84.8% (17,175/20,243) in 2014 to 91.2% (93,742/102,764) in 2016 and remained around 90% in each subsequent year. This corresponds with malaria post saturation in the target area, where villagers may have needed to travel to another village to receive diagnosis and treatment at the beginning of METF, but by 2016 most had a malaria post in their home village. By year and township, the greatest proportion of individuals from outside the village tested for malaria was observed in 2014 in Hlaingbwe (21.6%, 419/1940) and Hpapun (19.1%, 1767/9230). Additional file 5: Table S2 provides further details on residence by year and township. The test positivity rate was 50.5% higher for individuals tested outside their resident village than for village residents (16.7% compared to 11.1%).

### Impact on *P. falciparum *and *P. vivax*

In 2021, the average monthly *P. falciparum* incidence had declined by 89.3% from the start of the programme in 2014, and by 57.0% from when the near complete malaria post network was functioning in 2016 (Fig. 3). Between 2020 and 2021, the average monthly *P. falciparum* incidence increased by 67.0% in Hpapun (from 0.88 to 1.47 cases per 1000 person-months), while remaining stable in the other townships (Table 1).

**Table 1:** Average monthly incidence of *P. falciparum* and *P. vivax* by year and township

Township	2014	2015	2016	2017	2018	2019	2020	2021
	*P. falciparum* incidence per 1000 person-months							
Hpapun	13.99	7.41	3.88	3.71	3.17	0.86	0.88	1.47
Hlaingbwe	0.85	0.34	0.16	0.05	0.31	0.01	0.004	0.004
Kawkareik	0.26	0.16	0.03	0.09	0.09	0.01	0.00	0.003
Myawaddy	2.43	0.45	0.06	0.06	0.01	0.02	0.01	0.02
	*P. vivax* incidence per 1000 person-months							
Hpapun	12.81	5.80	4.60	6.81	9.78	7.93	7.07	13.01
Hlaingbwe	4.09	1.16	0.65	0.54	1.08	0.62	0.51	2.19
Kawkareik	0.58	0.56	0.14	0.13	0.26	0.10	0.05	0.05
Myawaddy	8.18	6.02	3.99	4.23	4.11	3.54	5.42	7.97

The same declines have not been observed for *P. vivax* incidence, which in 2021 had declined by 12.2% when compared to 2014 and had increased by 202.9% when compared to 2016 (Fig. 3). By township, Hpapun has seen the highest burden of *P. vivax* over the course of the programme (Table 1). An additional figure showing the incidence and testing rates from 2019 onwards provides another way of observing changes in incidence over time (see Additional file 5: Fig. S3).

Of the individuals diagnosed with *P. falciparum* and with information collected on their gender and age, 58.6% (10398/17739) were male, 16.4% (2916/17744) were under 5 years of age, 38.9% (6897/17744) were between 5 and 15 years of age and 44.7% (7931/17744) were older than 15 years of age. Of the individuals diagnosed with *P. vivax* with information collected on their gender and age, 58.8% (36746/62500) were male, 23.7% (14838/62538) were under 5 years of age, 41.6% (26026/62538) were between 5 and 15 years of age and 34.7% (21674/62538) were older than 15 years of age.

From 2020 to 2021, the yearly *P. falciparum* and *P. vivax* test positivity rates increased by 60.3% (from 0.63 to 1.01) and 78.1% (from 8.44 to 15.03), respectively, across the four townships. Despite a 34.8% increase in the *P. falciparum* positivity rate in Hpapun (from 1.35 positive per 100 tests in 2020 to 1.82 in 2021) this represents a 73.3% decrease compared to the *P. falciparum* test positivity rate in 2016 (6.81 positive per 100 tests) when the near complete malaria post network was established. In 2021, *P. vivax* test positivity rates were higher than in any previous year in Hpapun (19.6 positive per 100 tests), Hlaingbwe (6.4 positive per 100 tests), and Myawaddy (17.0 positive per 100 tests).

### Case investigations

**Fig. 4:**
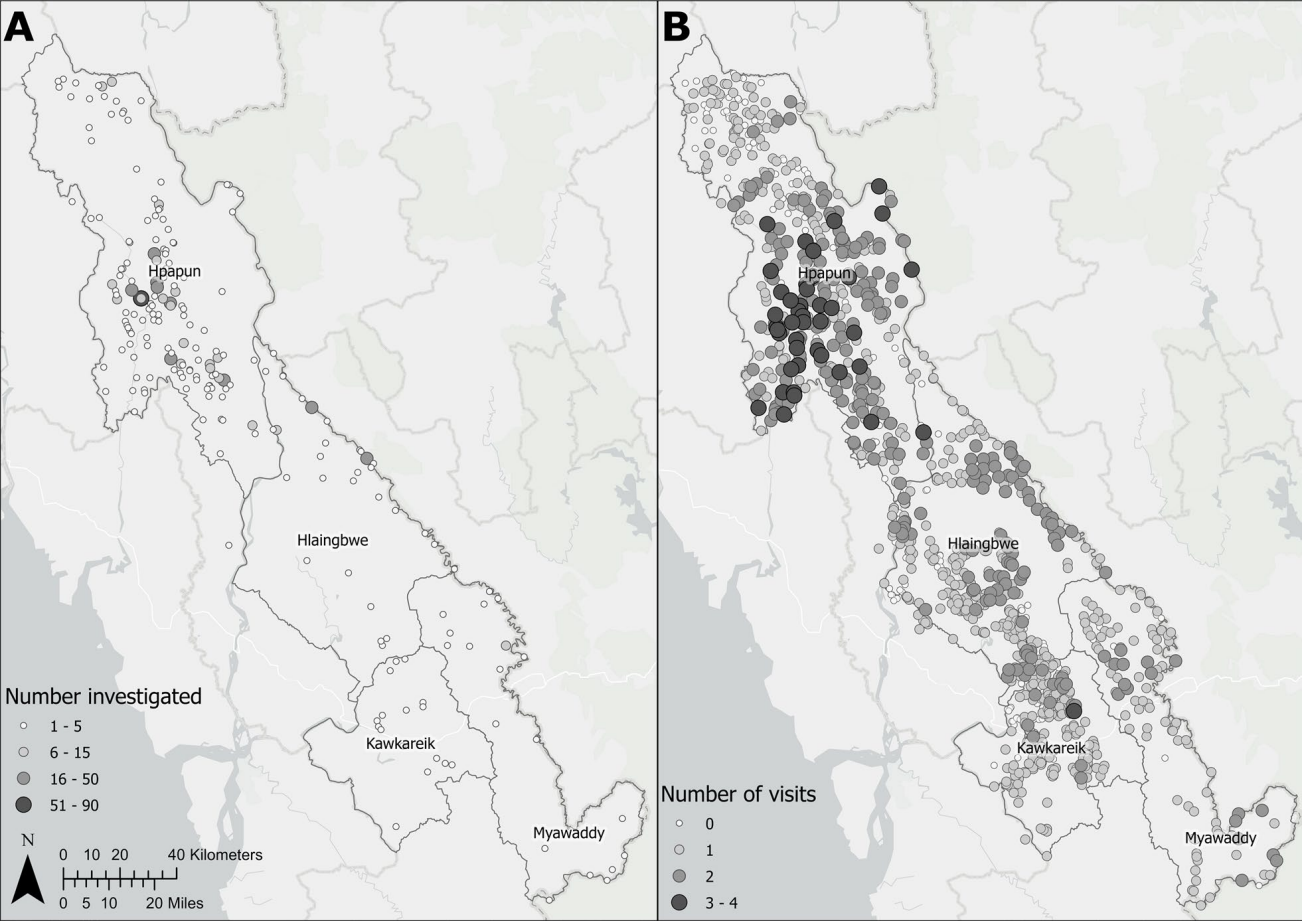
Number of **A** *P. falciparum* cases investigated, and **B** monitoring and evaluation visits per malaria post. All individuals diagnosed with *P. falciparum* that could be contacted were investigated in Hlaingbwe, Kawkareik, and Myawaddy from 2017, and in Hpapun from 2019. Monitoring and evaluation visits were conducted between 2017 and 2021 to assess malaria post performance based on predefined performance indicators. Maps generated using ArcGIS Pro version 2.5

The percentage of total *P. falciparum* cases investigated each year increased from 1.0% (35/3570) in 2017, to 69.1% (421/609) in 2020 (Table 2). Between 2017 and 2021, 10.5% (911/8667) of *P. falciparum* cases diagnosed at METF malaria posts were investigated. Figure 4 shows the spatial distribution of investigated *P. falciparum* cases.

**Table 2:** Percentage of *P. falciparum* cases investigated by year and township

Township	Year of case investigation % (n/N)				
	2017	2018	2019	2020	2021^†^
Hpapun	0.1 (3/3482)	0.0 (1/2817)	4.9 (34/694)	68.8 (413/600)	41.2 (310/753)
Hlaingbwe	23.5 (12/51)	40.5 (66/163)	91.7 (11/12)	100 (5/5)	0.0 (0/0)
Kawkareik	44.4 (4/9)	81.8 (9/11)	100.00 (9/8)*	− (0/0)	100 (1/1)
Myawaddy	57.1 (16/28)	87.5 (7/8)	85.7 (6/7)	75.0 (3/4)	7.69 (1/13)
Total	1.0 (35/3570)	2.8 (83/2999)	8.3 (60/721)	69.1 (421/609)	40.6 (312/768)

^†^ Data collection for 2021 ongoing

^*^ Additional case identified from the field not captured by the weekly data reporting system

#### Timing of diagnosis

The number of days between *P. falciparum* diagnosis and date of investigation varied by year and township. The median number of days between diagnosis and case investigation was 1 (25th–75th percentiles 0–7 days) for Hlaingbwe, 2 (1–11) for Myawaddy, 3.5 (0–13) for Hpapun and 7 days (1–16.5) for Kawkareik. Between 2018 and 2019, the median number of days between diagnosis and case investigation increased from 1 (0–6) to 12 (2–21.5), then decreasing in 2020 to 4 (1–12) and again in 2021 to 1 (0–6.75).

Of the 911 *P. falciparum* cases investigated, 61.1% (557/911) attended the malaria post after more than 24 h of fever (66.4% of consultations were within 48 h of fever onset). The most common reasons for delay were that they didn’t think it was serious (54.5%, 281/516), they didn’t think they had malaria (8.5%, 44/516) or they were too busy to attend the malaria post sooner (7.2%, 37/516).

#### Long-lasting insecticidal nets (LLINs)

Information on LLIN usage when at home was collected for 95.1% (866/911) of *P. falciparum* cases investigated. Overall, 33.8% (293/866) of individuals reported always using an LLIN whilst sleeping, 29.9% (259/866) reported sometimes using an LLIN whilst sleeping and 36.3% (314/866) reported never using an LLIN whilst sleeping. Over time the percentage of investigated *P. falciparum* cases reporting LLIN usage either always or sometimes whilst sleeping decreased from 73.6% in 2017 to 41.4% in 2018, largely influenced by a lower LLIN usage for cases investigated in Hlaingbwe in 2018 (decreasing from 77.8% in 2017 to 33.3% in 2018), but then remained at or above 65% in each subsequent year.

#### Travel

Information on time spent outside their resident village for more than 1 day in the 3 weeks prior to diagnosis was collected for 96.6% (880/911) of the cases investigated, of which 26.6% (234/880) reported travel outside of their resident village. By township, the highest percentage of cases reporting travel with an overnight stay prior to diagnosis was reported in Myawaddy (69.0%, 20/29), followed by Kawkareik (50.0%, 10/20), Hlaingbwe (29.4%, 25/85), and Hpapun (24.0%, 179/746).

Of the individuals who provided details on their travel, 47.9% (112/234) travelled up to 1 h away from their village (94 by walking, 18 by motor vehicle (tractor, motorbike, or car)), 29.9% (70/234) travelled between 2 and 3 h away (41 by walking, 29 by motor vehicle), and 22.2% (52/234) travelled 4 or more hours away (24 by walking, 26 by motor vehicle, 2 without details).

For those who stayed overnight away from their resident village and who provided details, the most common reasons for travel were farming or attending to livestock (48.9%, 67/137), and other work outside of the village (48.2%, 66/137). While sleeping outside of their resident village, 72.2% (164/227) did not sleep under an LLIN, and 70.2% (157/225) slept either outside or in a farm hut.

Information on travel outside of the village for daily activities (returning home the same day) in the 3 weeks prior to diagnosis was collected for 95.4% (869/911) of the cases investigated, of which 24.4% (212/869) reported travel. By township, the highest proportion of cases that had travelled for daily activities was reported in Hlaingbwe (47.0%, 39/83), followed by Myawaddy (34.5%, 10/29), Kawkareik (25.0%, 5/20), and Hpapun (21.4%, 158/737). Of the individuals who provided details on their travel, 88.0% (168/191) travelled up to 1 h away from the village (154 by walking, 12 by motor vehicle, 2 without details), 11.5% (22/191) travelled between 2 and 3 h away (17 by walking, 3 by motor vehicle, 2 without details), and 0.5% (1/191) travelled 4 or more hours away (by motor vehicle) before returning to their village. The most common reasons for travel were farming or attending to livestock (88.6%, 117/132).

Travel in the 3 weeks prior to diagnosis did not negatively impact treatment-seeking behaviour, where the reported day of fever at diagnosis remained between 2 and 3 days irrespective of travel. For individuals who travelled away from the village and returned the same day, 78.8% were diagnosed in their resident village. However, for those who travelled away from their resident village for more than 1 day (staying overnight), 56.9% were diagnosed in their resident village.

### Monitoring and evaluation visits

From 2016 to 2021, 1,390 monitoring and evaluation visits were conducted, covering 82.3% (1029/1250) of the complete malaria post network. By township, monitoring and evaluation visits were conducted at 82.1% (416/507) of malaria posts in Hpapun, 77.3% (309/400) in Hlaingbwe, 86.8% (203/234) in Kawkareik, and 92.7% (101/109) in Myawaddy. The majority (69.6%, 716/1029) of malaria posts were visited once, 26.2% (270/1029) were visited twice, and 4.2% (43/1029) were visited 3 or more times (Fig. 4).

Of the malaria posts visited, 66.2% (693/1047) were operated by one malaria post worker and 33.8% (354/1047) were operated by two malaria post workers, with 18 malaria posts reporting either an increase or decrease in the number of malaria post workers depending on village needs in a subsequent visit. The largest proportion of malaria posts with two malaria post workers were located in Myawaddy (75.3%, 76/101), followed by Kawkareik (65.5%, 133/203), Hlaingbwe (43.4%, 134/309), and Hpapun (2.6%, 11/416). This reflects the heterogeneity in village needs by township, where villages in Hpapun are smaller and so can be effectively managed by one malaria post worker, with a median household number of 28 (25th–75th percentile: 18–45). The median household number per village was closer to 70 in Myawaddy (72, 40–131), Hlaingbwe (70, 40–105), and Kawkareik (70, 50–103.75).

Indicators of the malaria post’s ability to provide uninterrupted access to diagnosis and treatment by year of monitoring and evaluation visit are presented in Table 3. The most common major dysfunction was malaria post closure for more than 24 h in the previous 2 months which was recorded in 25.0% (164/655) of all visits in Hpapun, 17.7% (21/119) in Hlaingbwe, 16.0% (63/393) in Kawkareik, and 15.7% (35/223) in Myawaddy.

**Table 3:** Summary of performance indicators of malaria post functioning recorded during monitoring and evaluation visits by year of monitoring and evaluation visit

	Year of assessment % (n/N*)					
	2016	2017	2018	2019	2020	2021
Total visits	161	558	447	140	77	7
*Major dysfunction*						
Malaria post worker absent	0.0 (0/161)	1.8 (10/558)	5.2 (23/447)	5.0 (7/140)	1.3 (1/77)	0.0 (0/7)
Malaria post closed for > 24 h in previous 2 months	22.4 (36/161)	25.1 (140/558)	20.1 (90/447)	10.7 (15/140)	1.3 (1/77)	14.3 (1/7)
Stock outs for > 2 days in previous 2 months	6.2 (10/161)	7.4 (41/551)	6.6 (28/426)	8.2 (11/135)	1.3 (1/76)	0 (0/7)
No valid^ψ^ RDT onsite	3.7 (6/161)	0.9 (5/557)	8.2 (36/437)	2.2 (3/139)	0 (0/77)	0 (0/7)
No valid^ψ^ ACT onsite	1.9 (3/161)	7.9 (44/556)	5.5 (24/437)	10.1 (14/139)	0 (0/77)	14.3 (1/7)
*Minor dysfunction*						
No regular financial support received from METF	3.7 (6/161)	2.2 (12/552)	0.7 (3/429)	0.0 (0/135)	0.0 (0/76)	0.0 (0/7)
Number of supervisor visits in previous 2 months						
0	19.0 (30/158)	35.8 (198/553)	35.5 (154/434)	57.3 (79/138)	22.4 (17/76)	0.0 (0/7)
1	18.4 (29/158)	13.0 (72/553)	10.1 (44/434)	6.5 (9/138)	4.0 (3/76)	14.3 (1/7)
2–3	12.7 (20/158)	24.4 (135/553)	17.5 (76/434)	13.0 (18/138)	34.2 (26/76)	57.1 (4/7)
≥ 4	50.0 (79/158)	26.8 (148/553)	36.9 (160/434)	23.2 (32/138)	39.5 (30/76)	28.6 (2/7)
No manual^†^ onsite	0.6 (1/161)	3.4 (19/556)	0.5 (2/444)	0.0 (0/140)	0.0 (0/77)	0.0 (0/7)
No reporting forms^‡^ onsite	0.6 (1/161)	1.4 (8/556)	3.6 (16/444)	2.1 (3/140)	0.0 (0/77)	0.0 (0/7)
No logbook^ξ^ onsite	31.7 (51/161)	52.5 (292/556)	2.9 (13/444)	2.1 (3/140)	0.0 (0/77)	0.0 (0/7)
No record of day of fever	2.5 (4/161)	4.0 (22/555)	5.2 (23/444)	17.1 (24/140)	2.6 (2/77)	0.0 (0/7)

^*^Some information could not be collected at every monitoring and evaluation visit resulting in different denominators

^ψ^ Invalid RDTs and artemisinin-based combination medicines are those that were expired or damaged and unusable

^†^Manual on guidelines for diagnosis and treatment response

^‡^Reporting forms used to summarize daily data at the end of each week

^ξ^ Logbooks used to record information for each case

For malaria posts where stock levels were inspected, 6.3% (86/1377) of malaria posts did not have valid artemisinin-based combination medicines in stock, and 3.6% (50/1377) did not have valid RDTs in stock at the time of assessment, where invalid artemisinin-based combinations and RDTs were classified as those that had expired or were damaged and unusable.

### Diagnosis and treatment knowledge

Between 2016 and 2019, 1491 treatment questionnaires were completed by METF staff. The majority (89.5%, 1335/1491) were completed by malaria post workers, followed by malaria post supervisors (9.3%, 138/1491) and Area or Zone coordinators (1.2%, 18/1491).

On average, the Area and Zone coordinators performed the best with an average correct response rate of 75.8% (range: 11–100%), followed by malaria post supervisors (71.4%, range: 6–100%), and malaria post workers (61.9%, range: 0–100%). Over the four-year period of knowledge assessment, the correct response rate increased for malaria post supervisors, from 67.8% in 2016 (range: 45–85%) to 85.6% (range: 60–100%) in 2019, and for malaria posts workers, from 55.2% in 2016 (range: 25–85%) to 61.2% (range: 15–100%) in 2019. Additional file 6: Table S3 provides a summary of the proportion of correct responses for each question on the questionnaire by malaria post worker staff.

## Discussion

Surveillance activities are essential to achieving malaria elimination, however the utility of surveillance data depends on how quickly it is collected, analysed, and used to action a response. This retrospective analysis of METF data describes the surveillance activities carried out by the programme, provides an assessment of data timeliness and completeness, and provides a summary of data collected from the different surveillance approaches used by the programme including weekly surveillance data used to monitor case trends, and case investigations.

In the METF programme, case investigations and targeted interventions were delivered in response to current malaria trends, necessitating the collection of real-time surveillance data. Between 2014 and 2021, the METF programme conducted MDA in 75 villages and MSAT in 22 villages in the METF target area in response to either a high prevalence (i.e. ≥ 40% prevalence of malaria, of which ≥ 20% of samples are *P. falciparum* positive) confirmed by quantitative polymerase chain reaction (qPCR) surveys [4], or an unexpected increase in case numbers. Targeted interventions were delivered on a case-by-case basis, based not only on current incidence but on incidence in the preceding months, information collected during case investigations and safe village access which has varied over time. From 2014 to 2016, late reporting in Hlaingbwe, Kawkareik and Myawaddy decreased, a result of the expanding cell phone network coverage over this period. In 2020, COVID-19 travel restrictions and village lockdowns were in place across Karen State [14, 16], resulting in an increase in late reporting for malaria posts in Hpapun where data must be physically transported from the malaria posts to data entry sites. In response to these delays, a more central data entry site was established in Hpapun, resulting in improvements in the timing of data availability and allowing for the continued monitoring of case numbers. In 2021, the military coup in Myanmar resulted in further disruptions to data transmission, particularly in Hpapun, where airstrikes and shelling limited the ability for safe movement between villages, and from malaria posts to data entry sites [16, 17]. Phone calls with malaria post workers have acted as an additional surveillance tool, particularly during these periods of disruption. Both improvements in timing of data availability, and the continued monitoring of malaria post activities with cell phone contact highlight the importance of a flexible surveillance system, remaining adaptive to changes in the region in which it operates.

Despite the impacts of both COVID-19 and the military coup, malaria testing rates in 2021 increased in Hpapun, Hlaingbwe and Myawaddy, likely a result of increasing *P. vivax* incidence. Cases of fever also continued to present to the malaria post early, with more than 80% of consultations occurring within 48 h of fever onset, thus preventing ongoing transmission [2]. The fact that testing rates, and the timing of consultations, were not negatively impacted by COVID-19 or the military coup demonstrates the importance of ensuring ease of access to malaria post services (through deployment of village-based malaria services) and continued engagement with the communities they serve [18]. Community perceptions and knowledge play an important role in treatment seeking behaviour [19–21] and highlights the need for continued community awareness campaigns and community engagement in ensuring the continued uptake and impact of malaria post services, even in the face of declining incidence [22].

From 2014 to 2017, the proportion of consultations with people from outside of the malaria post village decreased from 15.2% to 6.9% across the four townships, likely a result of the expanding malaria post network, delivering more localized and widespread access to malaria post services. In 2021, consultations in Hpapun by people from outside the malaria post village increased by 81.4% compared to 2020 (from 7.0 to 12.7%), likely a result of population displacement following the military coup.

Previous studies have discussed the impact of population movement on malaria transmission, on the risk of reintroduction, and on the spread of drug resistant parasites [7, 23–26], and others have highlighted the impact of population displacement on the emergence of malaria and other vector-borne disease outbreaks [27, 28]. From 2021, the military coup in Myanmar has resulted in population displacement across Karen State, and in 2020 and 2021, the METF programme was limited in its ability to provide targeted interventions to village-level increases in case numbers due to restricted movement and safety concerns when travelling. Moreover, the inability to provide safe curative treatment for *P. vivax* infections has limited the impact of early diagnosis and treatment on *P. vivax* incidence. It is, therefore, unsurprising that in 2021 there was an increase in the average monthly incidence of *P. falciparum* and *P. vivax* in Hpapun, and of *P. vivax* in Hlaingbwe and Myawaddy.

From 2017, *P. falciparum* case investigations were performed in Hlaingbwe, Kawkareik and Myawaddy, expanding to Hpapun in 2019 following a decline in *P. falciparum* case numbers. Of the *P. falciparum* cases investigated, 43.8% travelled away from their resident village in the three weeks prior to diagnosis, predominantly for farming or attending to livestock, returning the same day or staying overnight. However, without travel information prior to diagnosis of malaria negative individuals as well, associations between travel prior to diagnosis and positivity could not be investigated.

Despite the widespread delivery of LLINs [29], only a third of investigated *P. falciparum* cases reported always using an LLIN whilst sleeping in their resident village, and another third reported sometimes using an LLIN whilst sleeping. When sleeping outside their village only a third reported either always or sometimes using an LLIN. Previous studies on vector biting habits in four villages in METF study area, found only 36% of infective bites occurred indoors between 9 pm and 5am [30], with studies in other areas of Myanmar and the GMS reporting similar results [31, 32], emphasizing the importance of additional vector control measures, such as personal protection use when outdoors.

During monitoring and evaluation visits, malaria post performance was assessed using a set of performance indicators. The most common dysfunction identified was malaria post closure for more than 24 h in the previous 2 months. While this is an important indicator of malaria post functioning and must be monitored and responded to, people residing in these villages can still access malaria post services in neighbouring villages due to the wide coverage of the METF network. During monitoring and evaluation visits 35.0% of malaria posts reported no supervisor visit in the previous 2 months, however this does not mean no contact between the supervisor and the malaria post worker, as regular contact was needed for the transmission of data and stock replenishment which can occur away from the malaria post.

Between 2016 and 2019, questionnaires were used to assess malaria post worker knowledge of malaria diagnostics and treatment. While some gaps were identified in treatment knowledge for pregnant patients, generally malaria post workers performed well on questions essential in the correct treatment and response to malaria cases. On-the-spot retraining was provided following the completion of the questionnaire and retraining of malaria post workers was conducted each year, providing further opportunities for knowledge assessment and improvements in malaria post worker skills.

A limitation of this study is that not all *P. falciparum* cases could be investigated, so information was only available for the cases that could be traced, and the monitoring of changes in demographic details over time was limited. Additionally, the methods used for data collection and the responses available to indicators of malaria post functioning and increases in case numbers are context specific and may not be possible or suitable in other settings. However, the METF programme demonstrates that establishing a successful malaria elimination programme is achievable in settings with limited existing health infrastructure, and the key components to the programme’s success which include remaining adaptive to changes, building on existing health systems and social networks, working closely with local communities, the provision of early diagnosis and treatment, surveillance, and monitoring and evaluation are, and should be applicable in other settings. The study was also limited in the ability to quantify the impact of COVID-19 travel restrictions and the military coup in Myanmar on case numbers and availability of weekly surveillance data as the effects of both were heterogenous across space and the dates of COVID-19 village lockdowns remain unknown and vary across the townships as their enforcement was at the discretion of local authorities.

## Conclusion

Despite travel restrictions and village lockdowns in 2019 in response to COVID-19, and the ongoing military coup in Myanmar, which has resulted in widescale population displacement, there has been no reduction in malaria testing rates and malaria posts continue to provide early access to diagnosis and treatment in Karen State. In 2021, the military coup disrupted the usual data transmission routes, resulting in an increase in late reporting, however continued contact with malaria post workers has facilitated the ongoing collection of weekly surveillance data, *P. falciparum* case information and assessments of malaria post functioning. This highlights the importance of maintaining malaria services and a flexible approach to the collection and utilization of surveillance data, particularly in the face of reduced village accessibility.

## Supplementary Information

**Additional file 1. **Weekly data reporting form.

**Additional file 2. ***P. falciparum* case investigation form.

**Additional file 3. **Malaria post assessment form.

**Additional file 4. **Treatment questionnaire administered to malaria post workers, malaria post supervisors, and area and zone coordinators.

**Additional file 5. **Weekly surveillance and consultation data.

**Additional file 6. **Monitoring and evaluation assessments.

## Data Availability

The data analysed for this study are available upon request to the Mahidol-Oxford Tropical Medicine Research Unit data access committee: https://www.tropmedres.ac/units/moru-bangkok/bioethics-engagement/data-sharing.

## Abbreviations

METF: Malaria elimination task force
GMS: Greater Mekong Sub-Region
LLINs: Long-lasting insecticidal nets
G6PD: Glucose-6-phosphate-dehydrogenase
RDT: Rapid diagnostic test
ACT: Artemisinin-based combination therapy
MDA: Mass drug administration
MSAT: Mass screen and treat
