# Supplementary material for: Surveillance to achieve malaria elimination in eastern Myanmar: a 7-year observational study
Source: Malar J. 2022 Jun 7;21:175. doi: 10.1186/s12936-022-04175-w (PMC9171744; doi:10.1186/s12936-022-04175-w)
Supplement: Supplementary file 1 — Additional file 1. Weekly data reporting form. [file 12936_2022_4175_MOESM1_ESM.pdf]

**Additional file 1.** Weekly data reporting form.

| | |
|---|---|
| **MP Village Name:** \|______________________________\| | **MP (village) code:** \|___\|___\|-\|___\|___\|___\|___\|___\|  **Report by:** \|_____________________\| name |

## Malaria Elimination Task Force weekly reportform (SD BIOLINE)

***From date:**\|___\|___\| \|___\|___\| \|___\|___\| **to date:** :\|___\|___\| \|___\|___\| \|___\|___\|

*Day* *Month* *Year* *Please count from **MONDAY** to **SUNDAY** always

**Table 1:** Total fever cases (Number of cases of **FEVER**)

| | **Fever cases** |
|---|---|
| **<5 years** | |
| **5 to 15 years** | |
| **Older than 15 years (>15)** | |
| **Total** | |

**Table 2**: Laboratory results SD BIOLINE

| | PF | PV | Neg | Invalid | Total |
|---|---|---|---|---|---|
| <5 years | | | | | |
| 5 – 15 years | | | | | |
| Older than 15 years (>15) | | | | | |
| Total | | | | | |

**Table 3**: Number of patients **TREATED** for malaria

| | PF | | 'V | | Total |
|---|---|---|---|---|---|
| | Male | Female | Male | Female | |
| <5 years | | | | | |
| 5 – 15 years | | | | | |
| Older than 15 years (>15) | | | | | |
| Total | | | | | |

A. **Severe cases** within reporting week: \|______\| cases

B. **Death due to Malaria or suspected of Malaria** within reporting week: \|______\|cases

C. Total pregnant women within reporting week: Neg |____| PF |____| PV |____|

---

**Remaining number of SD BIOLINE** tests in stock: BIOLINE |______|
**Remaining number of COARTEM tablets** for PF treatment: |_____________| tablets
