# Supplementary material for: Surveillance to achieve malaria elimination in eastern Myanmar: a 7-year observational study
Source: Malar J. 2022 Jun 7;21:175. doi: 10.1186/s12936-022-04175-w (PMC9171744; doi:10.1186/s12936-022-04175-w)
Supplement: Supplementary file 2 — Additional file 2. P. falciparum case investigation form. [file 12936_2022_4175_MOESM2_ESM.pdf]

Additional file 2. *P. falciparum* case investigation form.

PF case investigation questionnaire

Area ____ Zone ____

MP code: ____

Village name: ____

RDT/DBS code: ____

Date of diagnose: ___ / ___ / ___

Interviewee: ____

Interviewer: ____

1. Date of interview *(day/month/year)* : ___ / ___ / ___
2. Name of patient: ____
3. Name of head of household: ____
4. Age: ____ Months: ____
5. Sex: ☐ Male ☐ Female
6. Address: ____
7. Presently living: ☐ in the village of MP ☐ village near by ☐ other (explain): -----------------------------

   7.(b) if village near by or other: name of village and of 2 closest village (*or places such as clinic, school…*):

   Name of village: ____ MP code (if available): ____

   Name of closest village 1 ____ MP code 1 (if available): ____

   Name of closest village 2 ____ MP code 2 (if available): ____
8. Have you been living here for more than 6 months? ☐ Yes ☐ No
9. Rapid Test (RDT) result: ☐ Pf ☐ Pv ☐ Pf+Pv ☐ Neg
10. Did you take DBS? ☐ Yes ☐ No
11. Treatment: ☐ Coartem ☐ Low-dose PQ ☐ Chloroquine ☐ Paracetamol ☐ Other ____
12. Reason for taking test: ☐ Feeling fever ☐ Feeling other symptoms ☐ Not feeling sick but want to check

    ☐ Contact from Pf case ☐ Participant in survey ☐ Participant in mass screening activity

    ☐ Other (explain): ____
13. What is your occupation? (*several answer possible*)

    (for children) ☐ Going to school ☐ Stay home ☐ Follow parents in activities

    (for adults) ☐ Farming ☐ Logging ☐ Soldier ☐ Daily worker ☐ Housewife

    ☐ Staff (all kinds)

    For all ☐ Other (*specify*) ……………………………
14. DOF: After starting to feel sick, how many days did you wait before coming to the MP? ____ days.
15. If DOF>1 day, what was the main reason for which you didin't go to MP as soon as you started feeling sick?

    ☐ MP is too far ☐ Too busy, I didn't have time

    ☐ I didn't feel that this is serious disease ☐ I didn't have fever

    ☐ I didn't know about MP at that time

    ☐ I refer to see someone else (health provider) first. If yes which one: ____

    ☐ Other reason: ………………………………………………………………………………
16. Did you take malaria treatment since you started feeling sick? ☐ Yes ☐ No ☐ Don't remember

    15.b if yes, where did you get it? ☐ MP/clinic ☐ Buy from shop

    ☐ Other provider (*specify*): ……………………………………………

    15.c if yes, which treatment? ☐ Paracetamol ☐ ACTs ☐ Traditional

    ☐ Don't know ☐ other (*specify*): …………………………………………

    15.d if yes how many day of treatment? ____ Days (if don't remember, use 999 code)
17. Did someone in your house have fever during last week? ☐ Yes ☐ No

(If yes, invite them to come to malaria post for fever testing as well)

18. Did you have malaria during the last year? ☐ Yes ☐ No ☐ Don't know
19. If yes/don't know, did you take a treatment with ACT then? ☐ Yes ☐ No ☐ Don't know

<u>Going away from the village during the 3 weeks before you started feeling sick</u>

20. Did you have activities away from the village and sleep away from the village during the 3 weeks before you started feeling sick? ☐ Yes ☐ No (*if no go to 28)*
21. if yes, how many days away in total? ☐ Less than 7 days ☐ 7-15 days ☐ 15 days and more
22. if yes, where? ☐ Village near by ☐ other village ☐ other place ______

21. (b) Name of village and of 2 closest village (*or places such as clinic, school…*):

Name of village: ______ MP code (if available): ______

Name of closest village 1 ______ MP code 1 (if available): ______

Name of closest village 2 ______ MP code 2 (if available): ______

23. How far? ☐ 1 hour or less ☐ 2-3 hours ☐ 4hours to 1day ☐ Further away
24. Going by ☐ Walking ☐ Tractor ☐ Motorbike ☐ Car ☐ Other ______
25. For what? (*several answers possible: If child following parents report parents activity*)

☐ Farming ☐ Rubber ☐ Taking care of livestock ☐ On duty

☐ Mining ☐ Logging ☐ collecting forest product (hunting charcoal)

☐ Studying ☐ Social welfare ☐ other (*specify*) ______

26. Where did you sleep?

☐ In a house ☐ Outside ☐ In a dorm ☐ In a farm hut

☐ Other (*specify*) ______

27. Did you use a bed net at that time? ☐ Yes ☐ No ☐ Sometime

<u>Daily activities around the village during the 3 weeks before you started feeling sick</u>

28. Did you have activities outside of the village and come back the same day? ☐ Yes ☐ No

27.(b) Name of village and of 2 closest village (*or places such as clinic, school…*):

Name of village: ______ MP code (if available): ______

Name of closest village 1 ______ MP code 1 (if available): ______

Name of closest village 2 ______ MP code 2 (if available): ______

29. How far? ☐ 1 hour or less ☐ 2-3 hours ☐ 4hours to 1day ☐ Further away
30. Going by ☐ Walking ☐ Tractor ☐ Motorbike ☐ Car ☐ Other ______
31. For what? (*Several answers possible*)

☐ Farming ☐ Rubber ☐ Taking care of livestock ☐ On duty

☐ Mining ☐ Logging ☐ collecting forest product (hunting charcoal)

☐ Studying ☐ Social welfare ☐ other (*specify*) ______

<u>Sleep in the village</u>

32. While you are in the village, did you sleep under a bed net?

☐ Yes, every night ☐ Yes, some night ☐ Never

33. How many people (including you) usually sleep under the bed net? ______ Persons
34. Did you have animals (cows/pigs/ goats…) sleeping under your house or around? ☐ Yes ☐ No ☐ Don't know
