# Supplementary material for: Surveillance to achieve malaria elimination in eastern Myanmar: a 7-year observational study
Source: Malar J. 2022 Jun 7;21:175. doi: 10.1186/s12936-022-04175-w (PMC9171744; doi:10.1186/s12936-022-04175-w)
Supplement: Supplementary file 3 — Additional file 3. Malaria post assessment form. [file 12936_2022_4175_MOESM3_ESM.pdf]

**Additional file 3.** Malaria post assessment form.

# Malaria post assessment

Village Name________________________ Township___________ District ________State______ Malaria Post Code____________________ HH number_______________

Village GPS coordinates: LAT: __________________ LONG: _____________________

Name of Malaria post worker (1) _____________________ (2) ______________________

TRAINING: Yes ☐ No ☐

RETRAINING: Yes ☐ No ☐

Name of MP Supervisor ____________________________________

MP worker not present ☐ Number of days since MPW away _____ Number of days until back ______ If not at post, where did the MPW go? ______________________________

**Assessment questions to MP workers (Ask to malaria workers directly)**

| | | Condition | **Comment/remark** |
|---|---|---|---|
| 1 | Was the MP closed for > 24 hours in last 2 months?<br><br>If MPW was available even if MP was closed, mention in remark | ☐YES NO | |
| 2 | Are there valid ACTs in the MP? | ☐YES ☐NO | |
| 3 | Are there valid RDTs in the MP? | ☐YES ☐NO | |
| 4 | Were there >2 days out of stocks (RDTs or ACTs) in the past 4 weeks?<br>_____________________________________________________<br>Adequate or sufficient medication and supplies (observe and check carefully)<br>RDTs =_____tests ACT=_____tabs CQ = _____tabs<br>PMQ =_____tabs Clindamycin | ☐YES ☐NO | If no, ask why |
| 5 | How are the results reported?<br>☐ SMS ☐ Paper ☐ Other (___________) | | |
| 6 | Does the MPW receive regular financial incentive? | ☐YES ☐NO | |
| 7 | Is there another MP in the village? | ☐YES ☐NO | |
| 8a | If YES, specify the supporting organization | | |
| 8b | If Yes, do you receive malaria data from them? | ☐ YES ☐ NO | |

| | | | |
|---|---|---|---|
| 9 | How often did you receive the visit of your MP supervisor in the last 2 months? ________ time(s) | ☐1 Per Month<br>☐<1 Per Month<br>☐>1 Per Month | |

MP: Malaria post; MPW: Malaria post worker; RDT: Rapid diagnostic test; ACT: Artemisinin combination therapy; CQ: Chloroquine; PMQ: Primaquine

**Assessment by Evaluator (Check - List)**

| | | |
|---|---|---|
| 1 | Is there a Malaria Post Manual in the MP? | ☐ YES ☐NO |
| 2 | Are there reporting forms in the MP? | ☐ YES ☐NO |
| 3 | Is there a logbook (daily recording of individual patients) in the MP? | ☐ YES ☐NO |
| 4 | Are the "Days of fever" recorded for each patient? (Review the daily record sheets) | ☐ YES ☐NO |
| 5 | Are there more than 5 consecutive days without activity in the logbook? | ☐ YES ☐NO |

Activity: malaria post consultations.

1. Comment or suggestions from malaria post worker

   ______________________________________________________________________________
   ______________________________________________________________________________
   ______________________________________________________________________________
   __________________________________________________

2. Comments or suggestion from the observer

   ______________________________________________________________________________
   ______________________________________________________________________________
   ______________________________________________________________________________
   __________________________________________________

Name: __________________________

Signature: ______________________

Date: __________________________
