# Supplementary material for: Surveillance to achieve malaria elimination in eastern Myanmar: a 7-year observational study
Source: Malar J. 2022 Jun 7;21:175. doi: 10.1186/s12936-022-04175-w (PMC9171744; doi:10.1186/s12936-022-04175-w)
Supplement: Supplementary file 4 — Additional file 4. Treatment questionnaire administered to malaria post workers, malaria post supervisors, and area and zone coordinators. [file 12936_2022_4175_MOESM4_ESM.pdf]

Additional file 4. Treatment questionnaire administered to malaria post workers, malaria post supervisors, and area and zone coordinators.

Treatment questionnaire

1.	What is the correct treatment for pregnant patient diagnosed with *P. falciparum* during 1st trimester of pregnancy?<br>a) Quinine + Clindamycin x 7 days (Q7C7)<br>b) Co-artem + Primaquine (COA3 + PQ 1 time)<br>c) Co-artem + Primaquine (COA3 + PQ 3 time)<br>d) Chloroquine (CQ3)
2.	What to do if a patient vomits the drug more than 1 hour after taking it?<br>a) Repeat the 1st dose again.<br>b) Don't need to repeat the 1st dose<br>c) Stop the treatment<br>d) Change the treatment
3.	What is the correct treatment for pregnant patient diagnosed with *P. falciparum* during and 3rd trimester of pregnancy (between 3 — 9 months)?<br>a) Co-artem + Primaquine (COA3 + PQ 1 time)<br>b) Co-artem only (COA3)<br>c) Chloroquine (CQ3)<br>d) Quinine + Clindamycin x 7 days (Q7C7)
4.	Primaquine should be given to<br>a) f trimester of pregnancy<br>b) 2nd trimester of pregnancy<br>c) Breastfeeding mother with child less than 6 months.<br>d) None of above is true
5.	Treatment of *P. falciparum* in a patient who is allergic to coartem?<br>a) Quinine + Clindamycin x 7 days (Q7C7)<br>b) Chloroquine<br>c) Primaquine<br>d) No treatment

6.	What are anti-malaria drug dosage calculations based on<br>a) Weight (kilogram — kg)<br>b) Weight (pounds — lbs)<br>c) Age (months)<br>d) Age (years)

7.	What is the correct treatment for a non-pregnant patient (age > 5 months) diagnosed with mixed infection (*P. falciparum* & *P. vivax*).<br>a) Co-artem + Primaquine (COA3 + PQ 3 time)<br>b) Chloroquine (CQ3)<br>c) Quinine + Clindamycin x 7 days (Q7C7)<br>d) Co-artem + Primaquine (COA3 + PQ 1 time)
8.	What is the correct treatment for breast-feeding mother with 5 months old child diagnosed with *P. falciparum*?<br>a) Quinine + Clindamycin x 7 days (Q7C7)<br>b) Co-artem + Primaquine (COA3 + PQ 1 time)<br>c) Co-artem + Primaquine (COA3 + PQ 3 time)<br>d) Co-artem only
9.	Treatment of *P. falciparum* in a child under 5 years (but > 6 months old)<br>a) Co-artem + Primaquine (COA3 + PQ 1 time)<br>b) Chloroquine (CQ3)<br>c) Quinine + Clindamycin x 7 days (Q7C7)<br>d) Co-artem only
10.	How will you treat a non-pregnant adult patient with no fever and *P. falciparum* RDT positive? a) Quinine + Clindamycin x 7 days (Q7C7)<br>b) Chloroquine<br>c) Co-artem + Primaquine (COA3 + PQ 1 time)<br>d) No treatment till fever develops
11.	Treatment of *P. falciparum* in a patient that cannot eat or drink<br>a) Refer the patient to nearest hospital or health centre<br>b) Try to give 1st dose of Co-artem and refer quickly to nearest hospital or health centre<br>c) Give Co-artem and primaquine (COA3 + PQ) and send back home d) No treatment

12.	Treatment of *P. falciparum* in child with fever who just wake up from a convulsion<br>a) Cool the child and try to give 1st dose of Co-artem and refer<br>b) No treatment<br>c) Refer the patient to hospital or health centre without treatment<br>d) Give Co-artem and primaquine (COA3 + PQ) and send back home
13.	Treatment of patient with a positive *P. falciparum* RDT 1 week after a <u>complete</u> treatment for malaria? a) Quinine + Clindamycin x 7 days (Q7C7)<br>b) Chloroquine<br>c) Repeat Co-artem<br>d) Give paracetamol and ask to come back if fever continues
14.	Treatment of *P. vivax* in a child who was treated 1 month ago for *P. vivax*? a) Chloroquine<br>b) Co-artem<br>c) Primaquine<br>d) No treatment

15.	Why do we need to treat *P. falciparum* within 48 hours of fever?<br>a) To stop the transmission of *P. falciparum* from one person to another<br>b) To stop the spread of malaria drug resistance in the area<br>c) To prevent the patient to become severe.<br>d) All of the above is true
16.	What to do if a patient vomits the drug less than 30 min after taking it?<br>a) Repeat the 1st dose again.<br>b) Don't need to repeat the 1st dose<br>c) Stop the treatment<br>d) Change the ACTs
17.	How do we administer Coartem for better absorption?<br>a) Water<br>b) Some fat (Milk or after meal)<br>c) Tea<br>d) Coffee

18.	What is the correct treatment for pregnant patient diagnosed with *P. vivax* in first trimester? a) Co-artem<br>b) Chloroquine (CQ3)<br>c) Chloroquine + Primaquine (CQ3 + PQ)<br>d) Quinine + Clindamycin x 7 days (Q7C7)
19.	Treatment of *P. falciparum* in adult<br>a) Chloroquine (CQ3)<br>b) Quinine + Clindamycin x 7 days (Q7C7)<br>c) Co-artem only<br>d) Co-artem + Primaquine (COA3 + PQ 1 time)
20.	What is the correct treatment for pregnant patient diagnosed with Mixed infection (*P. falciparum* & *P. vivax)* during 1st trimester of pregnancy (within first 3 months)?<br>a) Co-artem + Primaquine (COA3 + PQ 3 time)<br>b) Chloroquine (CQ3)<br>c) Quinine + Clindamycin x 7 days (Q7C7)<br>d) Co-artem + Primaquine (COA3 + PQ 1 time)
