# Supplementary material for: Surveillance to achieve malaria elimination in eastern Myanmar: a 7-year observational study
Source: Malar J. 2022 Jun 7;21:175. doi: 10.1186/s12936-022-04175-w (PMC9171744; doi:10.1186/s12936-022-04175-w)
Supplement: Supplementary file 5 — Additional file 5. Weekly surveillance and consultation data. [file 12936_2022_4175_MOESM5_ESM.docx]

**Additional File 5.** Weekly surveillance and consultation data.

**Table S1. Percent and proportion of weekly reports received late by year and township.**

| Township | 2014 | 2015 | 2016 | 2017 | 2018 | 2019 | 2020 | 2021^†^ |
| --- | --- | --- | --- | --- | --- | --- | --- | --- |
|  | Percent (%) (n/N*) | | | | | | | |
| Hpapun | 11.4  (273/2398) | 14.8  (1907/12907) | 15.0  (3349/22277) | 10.4  (2490/23844) | 24.7  (6169/24964) | 18.6  (4626/24877) | 29.6  (7061/23873) | 43.3  (8991/20766) |
| Hlaingbwe | 50.4  (461/915) | 20.7  (1155/5592) | 9.9  (1418/14354) | 11.6  (2266/19526) | 12.0  (2347/19557) | 10.2  (1953/19198) | 10.4  (1918/18508) | 19.3  (1649/8533) |
| Kawkareik | 72.2  (164/227) | 34.4  (810/2356) | 3.9  (325/8285) | 8.6  (975/11345) | 7.7  (890/11516) | 8.0  (934/11645) | 11.5  (1358/11818) | 8.0  (514/6411) |
| Myawaddy | 39.5  (716/1813) | 37.2  (1578/4246) | 22.6  (1182/5235) | 20.3  (1083/5330) | 13.7  (748/5456) | 13.4  (719/5364) | 28.2  (1536/5442) | 15.1  (665/4395) |
| Total | 30.2  (1614/5351) | 21.7  (5450/25101) | 12.5  (6274/50151) | 11.3  (6814/60045) | 16.5  (10154/61493) | 13.5  (8232/61084) | 19.6  (11873/60641) | 29.5  (11819/40105) |

Reports were considered late if entered more than 14 days from end of reporting week for malaria posts in Hpapun and more than 7 days from end of reporting week for malaria posts in Hlaingbwe, Kawkareik and Myawaddy.

*Total reports include only reports with date of data entry.

^†^ Year ongoing

**
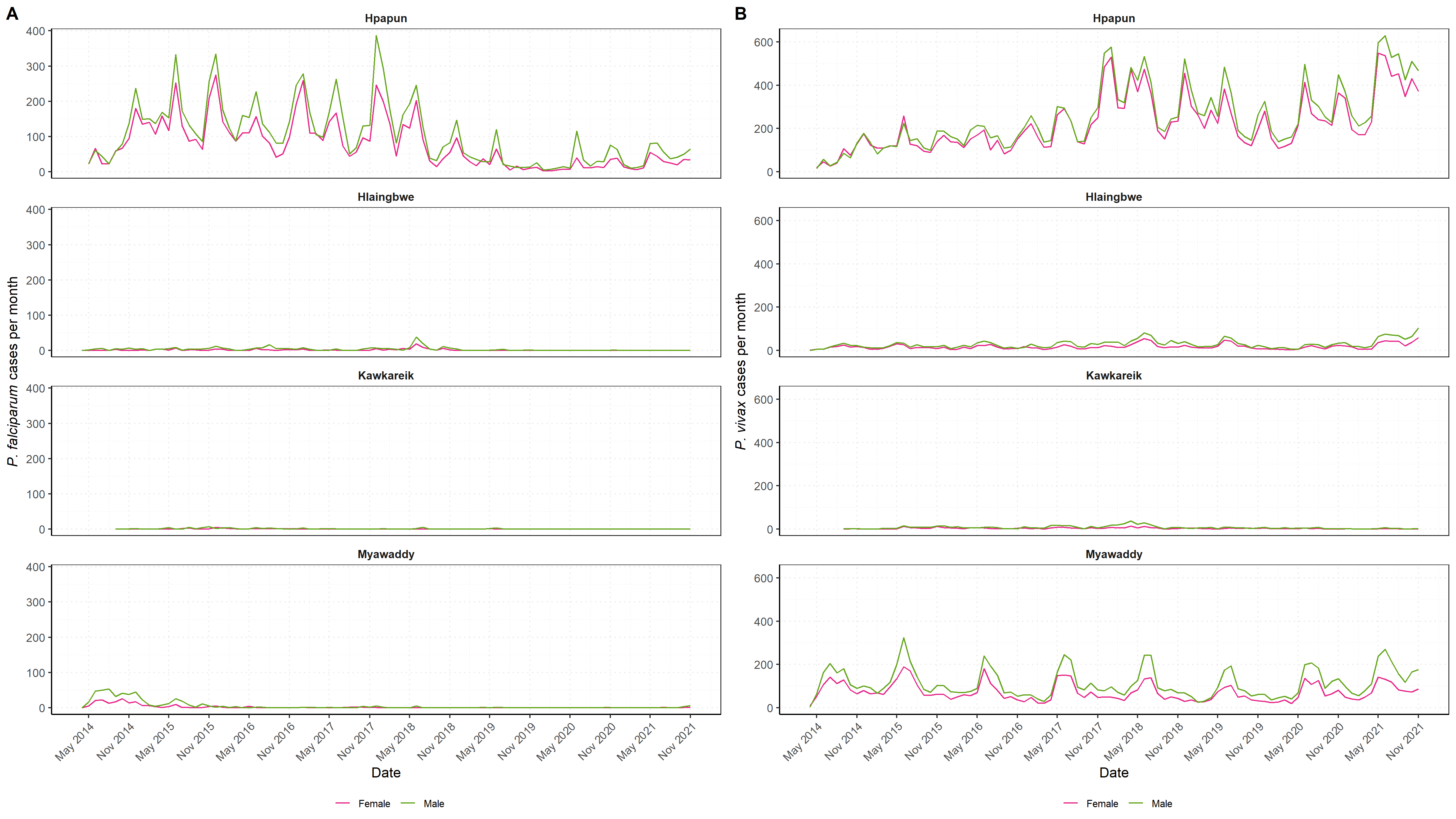
Figure S1. Number of females and males diagnosed with (A) *P. falciparum* or (B) *P. vivax* by date and township.** Total number of females (red line) and males (green line) diagnosed with *P. falciparum* or *P. vivax* at the METF malaria posts.

**
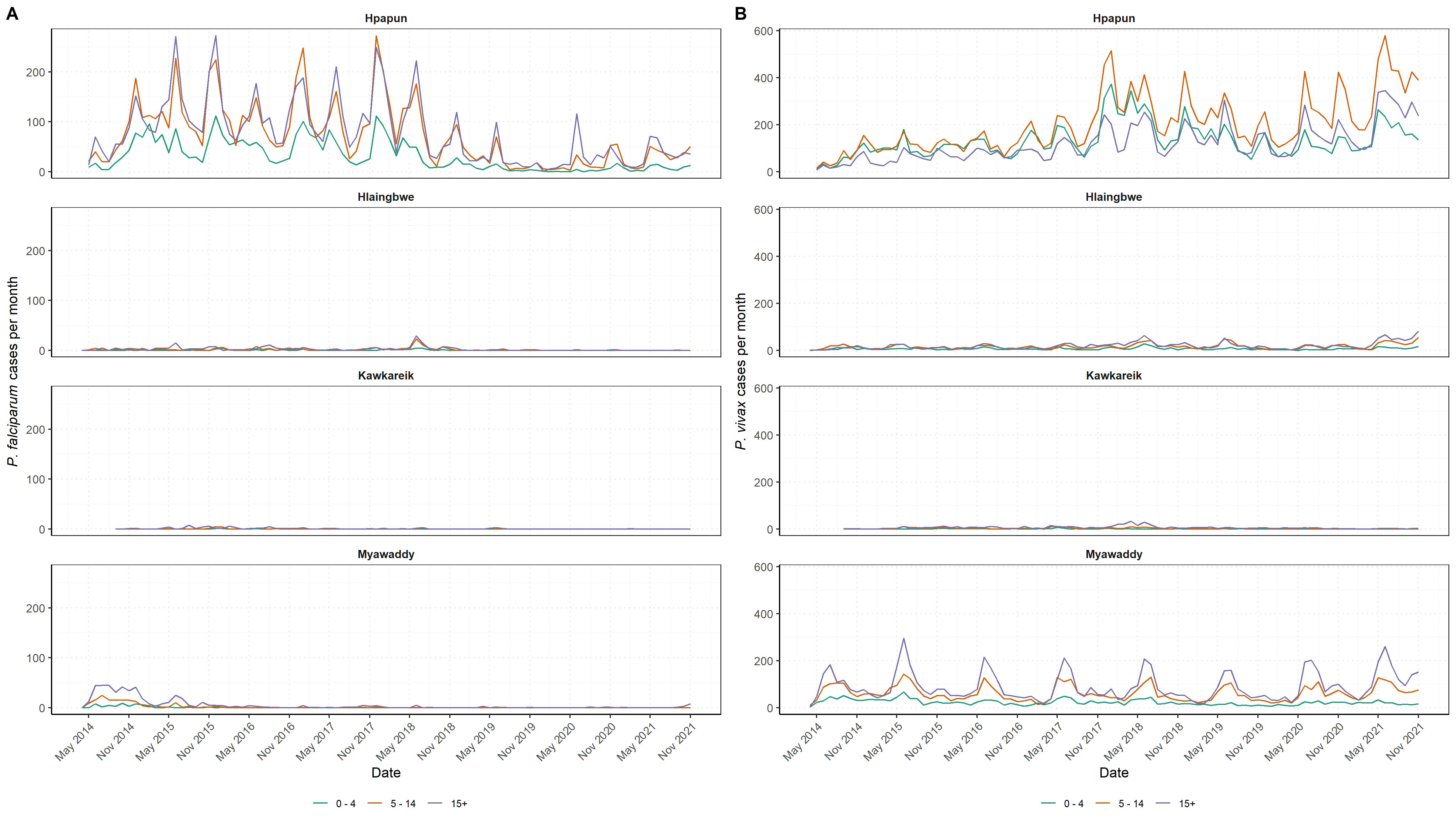
 Figure S2. Number of each age group diagnosed with (A) *P. falciparum* or (B) *P. vivax* by date and township.** Total number of individuals (less than 5 years of age – green line, between 5 to 15 years of age – orange line, and over 15 years of age – purple line) diagnosed with *P. falciparum* or *P. vivax* at the METF malaria posts.

**Table S2. Percent and proportion of consultations in people who typically reside in the malaria post village by year and township.**

| Township | 2014 | 2015 | 2016 | 2017 | 2018 | 2019 | 2020 | 2021^†^ |
| --- | --- | --- | --- | --- | --- | --- | --- | --- |
|  | Percent (%) (n/N*) | | | | | | | |
| Hpapun | 80.9  (7463/9230) | 83.6  (31423/37585) | 87.6  (39742/45357) | 92.9  (44754/48152) | 89.6  (43320/48339) | 93.5  (40794/43645) | 93.0  (41674/44834) | 87.3  (27539/31537) |
| Hlaingbwe | 78.4  (1521/1940) | 86.3  (10834/12552) | 93.7  (23980/25600) | 92.7  (25551/27562) | 87.0  (21005/24145) | 91.5  (22054/24114) | 93.9  (22675/24156) | 91.3  (12280/13443) |
| Kawkareik | 100  (257/257) | 95.8  (4244/4431) | 98.4  (12134/12330) | 96.8  (13356/13799) | 93.4  (11317/12121) | 94.7  (11937/12609) | 95.5  (12147/12717) | 96.2  (5775/6002) |
| Myawaddy | 90.0  (7934/8816) | 86.2  (18073/20967) | 91.8  (17886/19477) | 91.5  (17607/19233) | 91.3  (15474/16949) | 91.2  (14844/16278) | 93.0  (13887/14936) | 91.5  (10786/11781) |
| Total | 84.8  (17175/20243) | 85.5  (64574/75535) | 91.2  (93742/102764) | 93.1  (101268/108746) | 89.7  (91116/101554) | 92.7  (89629/96646) | 93.5  (90383/96643) | 89.8  (56380/62763) |

*Total consultations include only those with information on residence.

^†^ Year ongoing


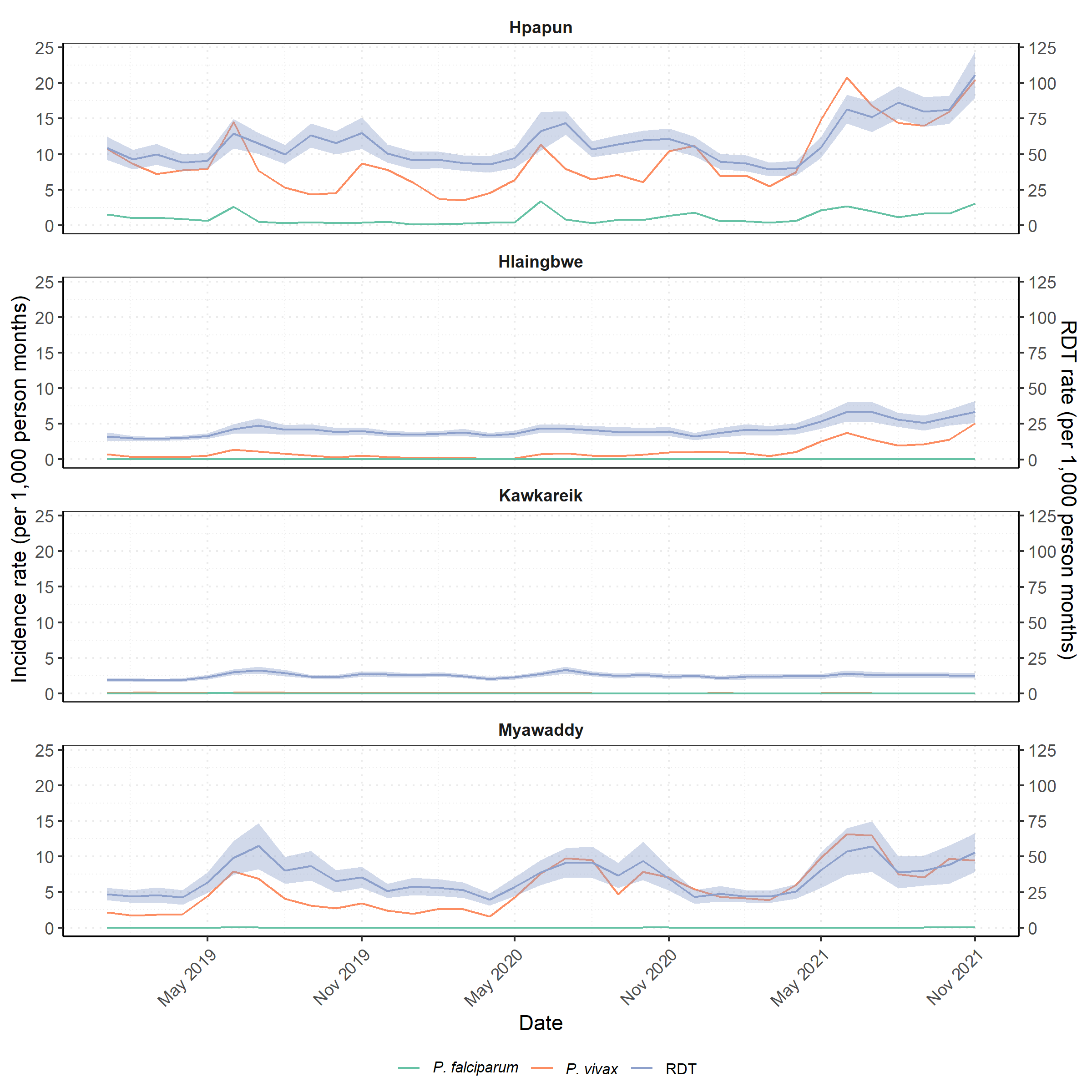


**Figure S3. Average monthly rate of RDTs and malaria incidence by date and township.** Average monthly rapid diagnostic testing rate (RDT – purple line) with 95% confidence intervals (purple area), and average monthly *P. falciparum* (green line) and *P. vivax* (orange line) incidence rates at the METF malaria posts from 2019 to 2021.
