# Supplementary material for: Surveillance to achieve malaria elimination in eastern Myanmar: a 7-year observational study
Source: Malar J. 2022 Jun 7;21:175. doi: 10.1186/s12936-022-04175-w (PMC9171744; doi:10.1186/s12936-022-04175-w)
Supplement: Supplementary file 6 — Additional file 6. Monitoring and evaluation assessments. [file 12936_2022_4175_MOESM6_ESM.docx]

**Additional File 6.** Monitoring and evaluation assessments.

**Table S3. Proportion of correct responses to treatment questionnaire by METF staff.**

| **Question** | **Malaria post worker** | **Malaria post supervisors, and area and zone coordinators** |
| --- | --- | --- |
|  | % (n/N) | |
| 1. Treatment for pregnant patient diagnosed with P. falciparum during 1^st^ trimester | 83.8  (1118/1335) | 86.5  (135/156) |
| 1. What to do if a patient vomits the drug more than 1 hour after taking it | 40.3  (538/1335) | 54.5  (85/156) |
| 1. Treatment for pregnant patient diagnosed with P. falciparum during 2^nd^ and 3^rd^ trimester of pregnancy | 47.3  (631/1335) | 53.9  (84/156) |
| 1. Primaquine should be given to | 69.2  (924/1335) | 82.1  (128/156) |
| 1. Treatment of *P. falciparum* in patient who is allergic to coartem | 47.0  (628/1335) | 69.9  (109/156) |
| 1. What are anti-malarial drug dosage calculations based on? | 92.2  (1231/1335) | 97.4  (152/156) |
| 1. Treatment for a non-pregnant patient (age > 5 months) diagnosed with mixed infection (*P. falciparum* and *P. vivax*) | 47.6  (636/1335) | 66.7  (104/156) |
| 1. Treatment for breast-feeding mother with 5 months old child diagnosed with P. falciparum | 47.9  (640/1335) | 58.3  (91/156) |
| 1. Treatment of *P. falciparum* in a child under 5 years (but > 6 months) | 50.1  (669/1335) | 53.9  (84/156) |
| 1. Treatment of a non-pregnant adult patient with no fever but *P. falciparum* positive | 75.1  (1003/1335) | 90.4  (141/156) |
| 1. Treatment of *P. falciparum* in patient who cannot eat or drink | 46.4  (620/1335) | 49.4  (77/156) |
| 1. Treatment of *P. falciparum* in child with fever who just woke up from convulsion | 60.5  (807/1335) | 63.5  (99/156) |
| 1. Treatment of patient with positive *P. falciparum* RDT 1 week after complete malaria treatment | 41.4  (552/1335) | 48.1  (75/156) |
| 1. Treatment of *P. vivax* in a child who was treated 1 month ago for *P. vivax* | 70.8  (945/1335) | 73.1  (114/156) |
| 1. Why do we need to treat *P. falciparum* within 48 hours of fever? | 58.4  (780/1335) | 84.0  (131/156) |
| 1. What do you do if patient vomits the drug less than 30 mins after taking it? | 75.8  (1012/1335) | 80.8  (126/156) |
| 1. How do we administer Coartem for better absorption | 85.7  (1144/1335) | 91.7  (143/156) |
| 1. Treatment for pregnant patient diagnosed with *P. vivax* in first trimester | 56.1  (749/1335) | 68.0  (106/156) |
| 1. Treatment of *P. falciparum* in adult | 75.0  (1001/1335) | 87.2  (136/156) |
| 1. Treatment for pregnant patient diagnosed with mixed infection (*P. falciparum* and *P. vivax*) during 1^st^ trimester of pregnancy | 66.3  (885/1335) | 78.9  (123/156) |
